# Observations on the Schools, Hospitals, and Practice of Italy

**Published:** 1829-06

**Authors:** G. A. Gordon

**Affiliations:** formerly House Surgeon to the Royal Infirmary, and President of the Royal Medical Society, of Edinburgh.


THE LONDON
### Medical and Physical Journal.

N° 364, VOL. LXI.] JUNE, 1829. [N° 36, *New Series.*

For many fortunate discoveries in medicine, and for the detection of numerous errors, the world is indebted to the rapid circulation of Monthly Journals; and there never existed any work, to which the Faculty, in Europe and America, were under deeper obligations than to the *Medical and Physical Journal of London,* now forming a long, but an invaluable series.—RUSH.

### ORIGINAL PAPERS, AND CASES,

### OBTAINED FROM PUBLIC INSTITUTIONS AND OTHER AUTHENTIC SOURCES.

### STATE OF MEDICINE IN ITALY.

*Observations on the Schools, Hospitals, and Practice of Italy.* By G. A. GORDON, M.D. formerly House Surgeon to the Royal Infirmary, and President of the Royal Medical Society, of Edinburgh.

No science has undergone so many and sudden changes as that of medicine, and on these have frequently depended the rise or fall of the reputation of medical schools. The day has not long gone by, when, the practice of medicine being conducted rather on empirical principles, than deduced from correct pathological knowledge, the medical schools of Italy enjoyed a higher reputation than those of England or France, and it was considered that a degree from the universities of Pavia and Padua, the most celebrated of Italy, was infinitely superior to that conferred by the universities of Edinburgh and Paris. The times are now, however, changed: the true value of anatomy and pathology is known; and, as these schools have made comparatively little progress in the cultivation of these fundamental parts of the healing art, their reputation has rapidly decayed, and now scarcely a stranger ever seeks a degree from schools whose honours were formerly so highly esteemed.

The MEDICAL SCHOOLS of Italy are numerous and extensive, and are all remarkable for a very strict academical arrangement. The clinical department, by far the most useful, in so far as it instructs young men in the practical part of their profession, is conducted in a most simple and judicious manner. The patients who form the subject of the clinical lectures are seldom more numerous than twenty, and are kept in a ward separate from the others. In several of the schools, as at Florence, each patient is intrusted to the care of a pupil, who observes minutely the nature of the disease, and the various changes which it undergoes, and makes an accurate report of them, which is read before all the other students, and commented on by the professor, at the bedside of the patient. The professor also gives a lecture daily, which includes a detailed account of each particular case. The limited number of clinical patients is a very great advantage; for, as the history and present circumstances of each are made fully known to the pupil, they are of much greater utility to him than if he were to attend to a crowd of patients, whose diseases he could only imperfectly understand. Those who have pursued the practice of the clinical wards of the Royal Infirmary of Edinburgh, or of the hospital of La Charité at Paris, and experienced the difficulties of understanding and selecting the cases, where the patients are so numerous, may easily conceive the truth of these observations.

All institutions are influenced in a great degree by the government of the country in which they are found. Under a despotic sway, although the general arrangements of universities may be the best possible, yet individual genius is sure to be cramped, and prevented from attaining that splendid and useful height to which, under freer and more favorable circumstances, it is generally seen to rise: and so it happens in Italy, where the arrangement of the medical schools has been fixed by a despotic legislature, and where every thing emanates from the hand of authority, that the professors, who, generally speaking, are men of very ordinary acquirements, seldom rise to their dignities by the force of talent, but are commonly indebted for their elevation to the interest and power of their families and patrons. The moment, too, a professor has attained, by whatever means, to the object of his ambition, he relaxes all his endeavours; for, as he receives the same stated salary whether his lectures attract few or many pupils, and as he has no prospect of rising higher, he has neither the stimulus of money nor ambition to push him forward in the road of improvement. In England, on the contrary, the attainment of a professorship, the value of which depends in a great measure on the reputation of the individual, is only an inducement to further honour and improvement; and thus it happens that, while the general arrangements and police of the Italian universities are the best possible, the professors themselves are deficient in the desire to promote the welfare of medical science, and consequently their lectures are devoid of that attractive interest necessary to captivate the minds of their hearers.

Of the despotic influence of the government the pupils also largely partake; for, accustomed as they are from their infancy to obey the mandates of authority, they are little suited, when they approach to manhood, and when acquiring medical information, to think for themselves, and to exert their own judgment on the subjects brought forward by the professor, uninfluenced by the particular tenets of any teacher: and thus, as the professor has no stimulus to improvement, and as the previous education of the pupil is such as to prevent him from maturing and strengthening his power of judgment, may be explained the cause, why, with such numerous and extensive schools, arranged too on an admirable system, the Italians, during a great number of years, have made so few improvements in medicine, and indeed have been unable to keep up with the rapid march of other countries.

It is indeed lamentable to see the apathy with which all improvements are regarded in Italy, and nothing struck me with greater astonishment, than the total want of originality of opinion among the pupils, who, with the blood of youth boiling in their veins, are content to follow implicitly the tenets of whoever leads them. The proud sentiment, "nullius addictus jurare in verba magistri," is unknown among them. At Edinburgh, which is certainly one of the best medical schools in Europe, (though as a surgical one it is, for obvious reasons, greatly inferior to many others,) a very different spirit pervades the minds of the students; and, although their independence of judgment may sometimes lead them astray, yet in general it has a beneficial tendency, and has frequently sown the germ of important discoveries, and first called into action the talents of some of the greatest men that have adorned the medical profession.

The great universities of Italy are those of Pavia, Padua, Pisa, Bologna, and Rome; and of these, Pavia has long been the most celebrated, and still enjoys the highest reputation. The system of education pursued in it is most complete. To obtain a degree in medicine, the candidate is obliged to have studied for a period of four years, during which he must have attended lectures on almost every branch of knowledge connected with the science of medicine, and particularly on anatomy and physiology, materia medica, and clinical and practical medicine, to which he devotes several courses. He must attend also lectures on surgery, clinical as well as theoretical; and is recommended to make himself acquainted with natural philosophy, natural history, geometry, &c.

The curriculum of the pupils in surgery is on the same extended scale. They devote their time principally to anatomy, physiology, theoretical surgery, clinical surgery, operations on the dead body, midwifery, &c. They also attend lectures on the more immediate subjects of medicine.

Before a student can be allowed to enter to any of these lectures, he must previously have undergone an examination in the more elementary branches of knowledge, such as Latin and Greek; and, at the commencement of each year, he is examined on the subjects embraced by the lectures of the last.

The examination for the degree of doctor is a public one. The candidate draws out of a bag containing the names of the principal diseases of the human body, those of four, which he delivers to the examinators, who immediately catechise him on every point connected with them. He is also shut up in a room, and writes a thesis on one of these diseases, solely from his own knowledge, and without the assistance of any books. The examinators then retire, and afterwards announce their determination to the public, as well as to the aspirant. Nothing can be a surer or fairer way of testing a candidate than this, and I apprehend that it might be adopted at Edinburgh and other schools with very great advantage, where it may have occasionally happened that a professor, going prepared beforehand, has taken undue advantage of the candidate.

After a student has obtained a degree, and before he can legally attend a patient, he must have followed the practice of a large hospital in the same capacity as our house surgeon and physician, for a period of two years; and afterwards have undergone an examination in practical medicine and surgery. Nothing can be more extensive and severe, and at the same time more fair and judicious, than this system of medical education, which is greatly superior to that adopted by any of the British universities.

The great ornament of this school, and indeed of Italy, is the veteran Scarpa, whose splendid talents have enriched, in an astonishing manner, the numerous subjects to which they have been applied. This great man, though now very far advanced into the vale of years, and nearly blind, continues to prosecute his researches; and the extensive correspondence which he maintains with men of talent in every quarter of the world, is a sure proof that he still lives to science. The subject of aneurism, with which his name is indelibly connected, is the one in which his mind is constantly engaged, and it is said he will soon give to the world some further observations on this interesting disease.

The university of Pisa,* situated in the Tuscan territory, is the one which ranks next to that of Pavia, though formerly Padua enjoyed as high a reputation as any. Pisa has been brought into notice principally by the talents of Vacca, whose premature death the medical profession has had so lately to deplore. The system of education at this school differs little from that adopted at Pavia. The most distinguished individual at present connected with it is Savi, the professor of botany.

The school of Bologna, indebted entirely for its present elevation to the exertions of Tommasini, attracts, for the study of medicine, a great number of pupils, but as a surgical school it is not in much estimation. The clinical instruction here is very extensive. A hospital, capable of containing a hundred patients, is appropriated to this purpose: half of these are, however, surgical cases.

Tommasini explains, at the bedside of each medical patient, every circumstance of his case, and afterwards gives a more general lecture at the university. The great number of clinical patients is rather, however, to be considered a disadvantage than an advantage to the pupils. The lectures of Tommasini, though highly theoretical, are yet replete with information, and are delivered in a very impressive and attractive manner. This great physician was obliged to fly from Parma, his native city, on account of his political writings, but his powerful talents soon procured him an extensive practice in his adopted city.

The university of Rome, called La Sapienza, as a general one is now probably the most complete and extensive on the continent, excepting that of Paris, but as a medical school it is inferior to those already mentioned. The building itself is a most magnificent structure; one, indeed, of the finest of modern Rome: it was designed by the famous

* I am perhaps mistaken in placing the Tuscan university before that of Bologna. It is difficult to determine that point, in consequence of individuals always supporting the reputation of that school with which they have been connected.

Michael Angelo. At this institution no less than forty-seven different lectures are given, the number of professors being forty-three. These lectures embrace, on a very extended scale, every branch of human knowledge. One of the most distinguished professors of this school is Morichini, professor of chemistry, the friend and associate of the illustrious Davy.

The hospitals of Italy are also numerous and extensive, but differ much in their police and arrangement. Some of them are admirably conducted, while others again are remarkable for filth, bad ventilation, unhealthy site, and crowded state. In all the hospitals there are a number of young men, from twenty to thirty, who perform almost the same functions as the dressers in the London hospitals, but live in the institution, and are lodged and fed at the expense of government. They also receive about three shillings a month of salary. Each patient, on his admission into an hospital, is provided with a robe, either red or black. The rest of his dress he must procure for himself. The beds in the Italian hospitals are in general formed of wood. The floors of the wards are all of brickwork, which is not washed oftener than once a year, but polished by means of constantly rubbing and brushing.

The principal hospital of Milan is a very large establishment, and well conducted; but none of its medical officers are men of great celebrity, and I observed nothing in their practice peculiar to them alone.

In Bologna, besides the clinical one already mentioned, there are three hospitals, extensive and well managed: from these the clinical hospital is supplied with the choicest cases, medical as well as surgical.

The hospital at Florence, which contains about four hundred patients, is one of the cleanest and best conducted in Italy, and attached to it is a small medical school. The system of clinical instruction here is well worthy of attention. The students are obliged to attend regularly every morning, their names being called over by the professor, who gives a lecture daily, and afterwards holds an examination. The professor of clinical medicine at this hospital, M. Nespoli, is one of the most intelligent physicians in Italy, and his attention and kindness to Englishmen I feel a grateful satisfaction in recording.

In Rome there are several hospitals, appropriated to different purposes: some of them, as the Consolazione, are excellently managed; while others, as the St. Jacomo, are exactly the reverse. The latter, capable of containing about six hundred patients, is intended for chronic surgical cases only. It is composed of two principal wards, a male and female; and two clinical rooms, which hold about seven patients each. The male ward is one of the most extensive I have ever seen, and contains, when full, nearly two hundred beds. These are arranged in four rows in the ward, which is not so broad as that of St. Bartholomew's hospital. The patients are kept in a very filthy state. The floors of the hospital are on a level with the ground, and the building is situated in a low and unhealthy part of Rome. From these circumstances it may be readily anticipated that the hospital cannot be a very healthy one; and accordingly I found that, at particular seasons, and especially in the dogdays, erysipelas and hospital gangrene raged to a very great extent. The latter attacks, at those seasons, every wound or ulcer, and frequently produces fatal consequences. The chief surgeon of this hospital is Sisco, who at one time enjoyed the first reputation in Rome, but is now too old to be an operating surgeon. He has done little for the advancement of surgical science. He has published only his Clinique, from which I was unable to gather any thing of importance. In it, however, he has given an account of a most enormous sarcomatous tumor growing from the neck, hanging over the shoulder, and reaching nearly to the level of the navel, which he extirpated with success. His most successful operations have been those for the stone, which he extracts by means of the lateral operation of lithotomy performed with a knife, much resembling that of Cheselden. The external incision he makes remarkably small. He is a great advocate against the tying of arteries in cases of aneurism, which he treats, and he alleges successfully, by means of compression. He has invented an instrument on the principle of the tourniquet, which he applies to the artery at some distance above the disease, so as to retard, though not completely to arrest, the circulation of blood through the aneurism. He afterwards applies compresses graduated over the tumor. The professor informed me that he had met with few cases which did not yield to this treatment; a statement which must be received with much qualification. In some instances where he had tied the artery, (previously to adopting this plan,) and particularly where he had followed the practice recommended by his celebrated countryman, Scarpa, of interposing a small compress between the artery and the ligature, he had encountered secondary hemorrhage. On the whole, he prefers amputating to the Hunterian operation. He prefers also tying the artery at the outside of the sartorius muscle, as being less likely to endanger secondary hemorrhage.

The Consolazione is devoted also entirely to surgical cases, such as accidents and sudden diseases requiring immediate operation. It is composed of two long wards, capable of containing one hundred patients each, one for males, the other for women. It is a remarkably clean and well-ventilated hospital, and affords quite a contrast to the other. It is under the charge of M. TRASMONDI, a very rough though excellent surgeon.

The medical hospitals of Rome are two; one, that of Santo Spirito, for males, and that of St. Giovanni for females. The hospital of Santo Spirito contains about seven hundred patients, and is under the direction of several men of talents and experience. The Clinique of this hospital, conducted by Professor MATHEIS, is one of the best to be met with any where. Professor FOLKI, a most intelligent physician, is also attached to this hospital.

The hospitals of Italy, like those of France, are entirely supported* by the government of the country, which has the entire management of them. No pupil ever pays any fee for being admitted to attend the practice. It would, it appears to me, be a great improvement in the hospital system of England, if these institutions were put under some general laws emanating from a committee of the House of Commons, or from any board of directors legislating for all: for it must be confessed that, even in the best-regulated hospital in England, some abuse, arising from private interest, is ever to be found; and in many of them abuses to a very great extent may be traced. It would also, I think, after having well considered the system of the continental schools, be a great advantage to science and the country, if all medical men, whether students or practitioners, were admitted free of expense, though subject to certain regulations, to see the practice of these establishments.

France, whose hospitals and medical schools are liberally open to the poor as well as the rich student, has derived singular advantages from the unrivalled and extended excellence of her medical men; and the vast improvements which they have introduced, particularly during the present century, into every department of medicine, sufficiently attest the benefits of the system.

* Voluntary contributions are, of course, frequently made to these institutions.

The enormous expense of attending the hospitals of England, and more especially of London, has generally been defended on the ground that it prevents low and uneducated men from attaining a sufficient knowledge of medicine to enable them to practise, and thus renders the profession more select and respectable. This reasoning, however, is selfish and erroneous; for the number of low, uneducated medical men is, I am confident, from my own experience, considerably greater than in France, while the system may be considered as the chief cause of the great number of unprincipled quacks who flourish in England. The disadvantages of it to science and the public are manifest and great. It is a notorious fact, proved by the history of mankind, that the greatest improvements have been introduced by those who have commenced from very humble beginnings; and a review of the history of the present most celebrated medical men in France accords with that statement. In London, the hospital surgeons and physicians are, in general, men of very extensive knowledge; but the great mass of practitioners, it will be admitted by every one at all conversant with the state of the medical profession in France, are greatly inferior in point of acquirements to their brethren in that country.

The MEDICAL PRACTICE of Italy is certainly greatly deficient in most of the grand points which are generally supposed to form the glory of English practice. An ignorance of, and inattention to, pathology is universal in Italy. Diseases are treated more according to the rules of systematic writers, than from a firm conviction of the dangers arising from a change in the structure of the organs of the body: symptoms are combated more than the actual disease; and hence it follows that the diagnosis of Italian physicians is frequently incorrect; their practice always feeble, often inert; and their remedies applied late, and with a sparing hand. From this general view of their practice, it may be readily conceived that their success is, in comparison with English practice of medicine, very limited; and I have little hesitation in stating that I have seen many patients die in the hospitals of Italy, who would undoubtedly have been saved by a more vigorous practice, such as that followed in England. Bloodletting, the grand sheet-anchor in acute disease, is used in a style throughout Italy, and more particularly at Rome, which must astonish every enlightened mind. Blood is drawn away in a small stream, in quantities from six to twelve ounces; the greatest care being taken to prevent *deliquium animi*, which is reckoned a very bad circumstance. These small bleedings are very frequently repeated, sometimes three or four times in the course of twenty-four hours, but, as may be well imagined, with little effect. In the course of an acute inflammation, the Romans perhaps abstract more blood than an English physician would do, but by no means with the same benefit; for it is an axiom in the practice of medicine of England, that a copious and well-managed venesection at the commencement of acute inflammation will be of more use, than numerous small ones during the progress of the disease.

The next most powerful remedy in subduing acute inflammation which we possess is the tartrate of antimony, and one which is in general estimation in England. The application of this remedy, in the treatment of inflammation, is little employed in Italy: at least, during my visit I never happened to see it used.* The kermes mineral, it is true, is in very frequent employment; but this more as a diaphoretic than employed in nauseating doses to depress the powers of the circulating medium. Decoctions of sarsaparilla and sassafras, and a variety of inert drinks, with numerous emollient clysters, are the means in general use. Purgatives to any great extent are not given; two or three grains of calomel being the strongest generally employed.

There is part, however, of the medical practice of Italy in inflammatory affections well worthy of attention, and that is the very strict attention to diet and regimen; a thing of the greatest importance in the treatment of all diseases, and one to which little attention is paid, comparatively speaking, in England.

Percussion and the stethoscope, the use of which has undoubtedly paved the way for those great discoveries of the pathology of the pectoral viscera, for which the world is indebted to the indefatigable talents of LAENNEC, are never employed in the hospitals of Italy; and, consequently, the Italian physicians are unable to form that accurate diagnosis of the various shades of disease to which these organs are so liable, and from which alone can be deduced a proper and certain method of cure.

It is very generally supposed in England that, owing to the mild and salubrious climate, phthisis pulmonalis is a

* Mr. NORTH has referred me to the Medico-Chirurgical Review for March 1822, p. 752, in which it is said that Dr. CARLO BELLATTI, a distinguished physician at Pavia, was in the habit of giving enormous doses of the tartar emetic: half a drachm to three drachms daily. This is certainly, however, not the general practice in Italy at the present moment.

very rare disease in Italy. This, however, is a very ill-founded opinion; for in every part of that country it is a very common complaint, and differs in no degree from that which is so great a scourge to the British isles.*

Fevers of every kind are numerous in Italy, and differ in their type according to the season of the year, but in general are complicated with affections of the chylopoietic and assistant chylopoietic viscera. Their treatment differs in little from that followed in England, and is in general very successful.

Italy is the country to study intermittent fever, which, during the summer and autumn, rages to a very great extent, and numerous cases of which, under a secondary form, are always to be found in the hospitals, even during the most wintry season of the year. The Pontine marshes, situated between Rome and Naples, are the most fruitful source of this form of fever; and during the season the hospitals of Rome, as well as those of Naples, are filled with cases produced by exposure to the malaria of these marshes. The spleen is, in general, the organ most affected. The liver, also, is very frequently attacked.

Some very excellent observations have been published on the composition of the atmosphere of the Pontine marshes, and particularly by M. Brochi, in conjunction with Professor Morichini; from which it appears that these excellent chemists were unable to detect any change in the composition of the air, except the addition of some aqueous exhalation in the worst places, and at the worst seasons of the year and periods of the day. From these observations, and those of Professor Folki, of Rome, it is also shown that an uniformly dry and warm state of the atmosphere is the freest from fever, and that the most numerous cases occur when rains are succeeded by intense heat.

The worst periods of the day are immediately before sunrise and immediately after sunset. Sleeping in the "mal'aria" is almost certain to induce intermittent fever. Woollen clothing, as flannel, is stated to be a great preservative against taking the fever; a fact well known to the ancient Romans.

Professor Folki denies, in toto, the existence of any peculiar obnoxious principle in the atmosphere of marshy grounds, and observes, that he considers the disease to be sufficiently accounted for, by the aqueous vapour condensing and producing cold, by the fall of the temperature of the

* In the Papal territory it is considered to be an infectious disease, and the police are extremely strict in burning the clothes of the dead, and in fumigating their apartments.

atmosphere at sunset, and by the cold wind, which frequently prevails, acting on a system out of order from irregularity of living or other causes. The Professor, who has been long engaged in researches into every point connected with this fever, considers the proximate cause of it to be a want of equilibrium between the electricity of the atmosphere and that of the body; a theory which he has sustained in a very plausible manner, though the reasoning rests rather on negative than positive evidence.

In an admirable clinical lecture by Professor Matheis on this subject, at which I was present, he observed that he had never seen a well-marked instance of the disease produced for the first time during the cold months of the year, and he was inclined to deny its possibility altogether. But during my stay at Rome I had an opportunity of seeing a well-marked instance. Dr. Fleming, of Manchester, was exposed, on the 12th of December last, the weather being at the time cold, to the influence of the malaria of the Pontine marshes. He was kept in the open air for an hour at midnight, at Teppacina, a small town at the frontier of the Roman territory, while his baggage was examined by the revenue-officers. He experienced at the time a sensation of cold, and next day had a most severe and well-marked attack of fever, which assumed the quotidian type, and was an extremely severe case. He suffered from it, more or less, for three months. I narrated this case to Professor Folchi, and he observed that he conceived it not impossible that a foreigner, unused to the air and climate of Italy, might be attacked even during winter; but he had, during his long acquaintance with the disease, never seen a single primary case during winter in a native of the Papal dominions.

Bark and sulphate of Quinine are the only remedies ever employed in Italy in the treatment of intermittent fever, and they are considered as specifics. The former, to the extent of half an ounce per day, is given in hospital practice, as being more economical; the latter, to the extent of six or eight grains a day, to private patients, as being a more agreeable, and perhaps more efficacious, form. Arsenic and opium, I was informed, are never employed during any stage or shape of intermittent fever. Secondary attacks of intermittent fever are not uncommon during the cold weather, and I saw numerous instances in the hospitals.

Surgical practice.—The general surgery of Italy, during later times, has made but little improvement, notwithstanding the writings of Scarpa and Vacca, and the facility with which the best authors of England and France are procured. The grand defect in Italian practice is the ignorance of the surgeons of the laws of nature in reference to the healing of wounds; laws which undoubtedly ought to be the basis of practical surgery, and a knowledge of which, first completely developed by the masterly genius of JOHN BELL, has raised English surgery to the eminent and successful state in which it is at present found.

The surgeons of Italy are still sadly ignorant of the facility of curing wounds, whether arising from accident or surgical operations, by what is called the first intention; and this may be considered the grand defect of Italian surgery, and one which pervades the whole practice. Thus, whether a mamma be extirpated or a tumor excised, the wound is stuffed with lint, and the patients, if they escape the violent inflammation which too often follows, are subjected to an extensive suppuration and long process of cure. All ulcers in Italy are dressed in the same way; that is, covered with charpie. The method of BAYNTON, the application of stimulants, and the use of constitutional means, as far as I could observe, are little, if at all, employed. The lunar caustic is sometimes employed to check granulations; but this is the only substance I have seen used in any of the hospitals of Italy. In amputations, however, it must be allowed, they adopt the English system of union by the first intention. At Rome, and in general throughout Italy, the circular amputation, "*en deux tems,*" is the one employed. The following is the method: A tourniquet (and those instruments in Italy are of a very miserable description,) is applied to compress the artery, at a very short distance indeed above the disease for which the amputation is performed. (This is a very great defect; for it frequently happens that the surgeon performs his incisions in diseased parts; or, if they are sound, he leaves too little to cover the bone.) A ribbon is then placed round the limb, to mark the point where the incision is to commence, and, by compressing the nerves, to prevent the pain as much as possible. The Italian surgeon, then, with a *large scalpel*, performs the circular incision in the usual way. (The scalpel is another great defect.) The ribbon is then withdrawn, and the muscles divided. The surgeon then ties the artery; and this is managed* in a style quite abhorrent to the genius of English surgery: a broad ligature is employed, and artery, veins, and a large portion of the muscles, are included. (The frequent consequences of such

* I here speak more particularly of the practice at Rome.

treatment need not be exposed.) The edges of the wound are then brought into contact by means of adhesive plaster.

There is one small circumstance connected with the dressing of wounds, which struck me as particularly neat, and which is perhaps of considerable importance, viz. the method of using adhesive straps. The strap is cut very broad at the extremities, and narrow in the centre, so that it is enabled to take a firm hold of the edges of the wound, and exert a considerable power in retaining them in contact, while large spaces are left between each slip in the middle, which permits the free discharge of the pus and ligatures.

This is a practice which, I apprehend, might be adopted in many cases of wounds in England, and particularly in those caused by the great operations of surgery, with very considerable advantage.

The Italian treatment of injuries of the head is in many cases too active, while in others patients are often lost from deficiency of vigor. Thus, in many of the hospitals of Italy, the system of trepanning, nearly as recommended by Pott, is pursued; but in patients attacked with that very common and insidious inflammation of the membranes of the brain, which is the result of external injury, and where the most vigorous depletory treatment is required, their measures are in general limited to one or two small bleedings, to the exhibition of a few purgatives, and the employment of drinks and decoctions of no avail.

Fractures of the extremities, in most of the hospitals of Italy, but particularly in those of Rome, are treated in the straight position. M. Trasmondi, one of the principal surgeons of that capital, informed me that his success was very great, and that he had come to the determination, from the results of an extensive practice, of employing that position in every instance. But nothing, surely, can be more erroneous or empirical than to lay down a general rule for the treatment of all cases, of whatever kind. In many instances of fractures of the inferior extremities, the perfectly straight position may undoubtedly be employed with the best success; but in many others, and indeed by far the greater proportion, the flexion of the knee is infinitely preferable: such, at least, is the opinion of the great hospital surgeons of England and France.

The number of cases of erysipelas, in some of the hospitals of Italy, is very great. They are treated invariably on the antiphlogistic system. In severe cases of the acute phlegmonoid erysipelas, bleeding, to the extent of eight or ten ounces, is practised, and purgatives and the kermes mineral are exhibited. The stimulating plan is, I believe, never employed; and Mr. Lawrence's method of incision is unknown. Where gangrene, however, has supervened, the Italian surgeons frequently make small scarifications, with the view of favoring the separation of the dead parts from the living. Their practice, as will be readily admitted by every enlightened surgeon, is founded on correct views of the disease, and is eminently successful.

Hospital gangrene, which is now rarely seen in England, in some of the hospitals, and in particular in that of St. Jacomo at Rome, is not uncommon, and produces the same train of fatal symptoms which marks its progress under every circumstance and in every clime. In the hospital of St. Jacomo it rages frequently to a considerable extent, particularly during the heat of summer; and even during winter I had an opportunity of seeing one or two instances of it. The Italian treatment of this disease consists in nourishing, supporting, and stimulating the system by every means in the power of the surgeon, by the exhibition of wine, opium, bark, sulphate of quinine, and other tonics. The wound is dressed simply with lint.

In the treatment of aneurism, the Italians seem to be exceedingly far behind. Their surgeons are timid, and unwilling to adopt those bold but successful operations which have shed such a brilliant lustre over the English surgery of the present century. During the time I spent in Italy, I did not hear of a single case where even the simpler operations for aneurism had been adopted; and many of the more difficult ones, but which are yet successfully employed in England, are unknown. Thus the ligature of the subclavian and external iliac arteries, operations which (it may almost be said) are in daily use in England, have never yet been attempted in Italy. The treatment of aneurisms consists commonly in palliative means. Compression is the most powerful of these, and it must be allowed is often employed with great advantage, and occasionally so as to effect a complete cure. It appears to me that this remedy is greatly too much neglected in England, and that surgeons, led away by the *éclat* of brilliant operations, do not pay sufficient attention to more simple and less dangerous means, and which are yet often completely successful. The method of Professor Sisco, already alluded to, is that generally adopted. I had an opportunity of seeing three cases of aneurism, under the care of M. Flajani, in the hospital of Santo Spirito, treated in this way, and which seemed to be undergoing a process of cure. They were all incipient forms of the disease.

The method of tying arteries recommended by the veteran SCARPA, has now fallen into disuse.

Different operations are performed for lithotomy in the different hospitals of Italy. In Tuscany, and particularly at Florence and Pisa, the barbarous operation of the gorget is in general use. At Milan, the bistoire cachée of the French is commonly employed; and at Rome the simple operation with the knife, nearly as practised by Cheselden, is that adopted. In all these schools, the surgeons agree in one point, that of making the external incision remarkably small. The fatality of the operation is said to be about three deaths in twenty throughout Italy.

If the stone be large, the recto-vesical operation of Vacca is preferred to the high operation, which is at present scarcely ever practised in Italy. The operation of M. Civiale for "la broiement de la pierre," and which certainly in a great number of patients may be employed with advantage, has not yet been introduced into use in Italy. In cases of stone in the female bladder, the operation by incision is adopted in preference to that by dilatation.

The operations for hernia are performed in Italy nearly in the same manner as in England, with this difference, that they are commonly performed too late. Two cases came under my notice at Rome, where a strangulated inguinal hernia had existed for three or four days when the patients were admitted into the hospital. The operations were not performed, in one, for twenty-four, and in the other for thirty-six, hours after, and in both the gut was found gangrenous, and death ensued.

The venereal disease, which bears in Italy precisely the same characters which mark its progress in England, is treated in a very mild, judicious, and efficacious manner. In cases of primary sores, calomel and blue pill are exhibited in small doses. When the constitution is tainted, the oxymuriate of mercury is given to the extent of the sixth of a grain for a dose.* These preparations are administered with much care and caution, and are never continued to such a length of time as to injure the constitution. Decoctions of sarsaparilla and sassafras, and other herbs of a similar nature, are given in conjunction with them, and the patient is enjoined a moderate, and frequently milk, diet.

Cases of venereal disease are by no means so common as

* This is particularly praised by Professor TAGLIABO.

in England; nor have I ever seen, in any part of Italy, such extensive ravages from the disease as in Britain.* In general the regulations of the police are extremely severe against women contaminated with this disease, and in the papal dominions no prostitutes are permitted; the moment females are discovered exercising this infamous profession, they are thrown into prison.

---

### MESMERISM.

* I ought to mention that I have not visited Naples, where the disease is said to assume a particularly severe form, and where some have asserted it first appeared. Throughout Italy it is termed "la malattia Francese;" thereby pointing out the source whence it came into this country.

